# Determinants of exclusive breastfeeding in an urban population of primiparas in Lebanon: a cross-sectional study

**DOI:** 10.1186/1471-2458-13-702

**Published:** 2013-07-31

**Authors:** Haya Hamade, Monique Chaaya, Matilda Saliba, Rawan Chaaban, Hibah Osman

**Affiliations:** 1 Department of Pediatrics and Adolescent Medicine, Faculty of Medicine, American University of Beirut, Beirut, Lebanon; 2 Department of Epidemiology and Population Health, Faculty of Health Sciences, American University of Beirut, Beirut, Lebanon; 3 Department of Health Promotion and Community Health, Faculty of Health Sciences, American University of Beirut, Beirut, Lebanon

**Keywords:** Breastfeeding, Postpartum support, Lebanon

## Abstract

**Background:**

The proportion of mothers who exclusively breastfeed their babies up to 6 months remains low. Determinants of breastfeeding practices have been largely documented in high-income countries. Little evidence exists on possible predictors of breastfeeding behaviors in the Middle East. Our aim was to assess the prevalence of breastfeeding in Beirut and determine the factors that impact breastfeeding behavior in this population.

**Methods:**

Data for this longitudinal study is nested within a randomized controlled trial (RCT) assessing the impact of a 24-hour hotline and postpartum support film on postpartum stress. Healthy first-time mothers delivering in the capital Beirut between March and July 2009, were interviewed at 1–3 days and 8–12 weeks post delivery. A multiple logistic regression analysis was used to determine the factors associated with exclusive breastfeeding at 8–12 weeks postpartum.

**Results:**

The overall breastfeeding rate at 8–12 weeks postpartum was 67%. The exclusive breastfeeding rate was 27.4%. Factors associated with exclusive breastfeeding included maternal work (OR=3.92; p-value<0.001), planned pregnancy (OR=2.42, p-value=0.010), intention to breastfeed (OR=3.28; p-value=0.043), source of maternal emotional support (OR = 1.87, p-value=0.039) and the use the postpartum support video, the hotline service or both (OR=2.55, p-value=0.044; OR=3.87, p-value=0.004 and OR=4.13, p-value=0.003).

**Conclusions:**

The proportion of healthy first-time mothers who exclusively breastfeed in Beirut is extremely low. Factors associated with breastfeeding behavior are diverse. Future research and interventions should target different levels of the maternal-child pair’s ecosystem.

**Trial registration:**

ClinicalTrials.gov, NCT00857051

## Background

The health benefits of breastfeeding have been widely cited. Breastfeeding has been shown to protect infants against common acute childhood infections, enhance the immune system, decrease rates of Sudden Infant Death Syndrome (SIDS), promote cognitive development and prevent chronic diseases such as obesity, diabetes mellitus (type 1 and 2), asthma and certain pediatric malignancies [1]. It also protects mothers against breast and ovarian cancer, osteoporosis and postpartum bleeding while promoting maternal-child bonding [2]. Furthermore, there is extensive evidence to support the psychosocial, economic, and environmental benefits of breastfeeding. In the United States (US), these benefits include the potential to cut 3.6 billion dollars in annual health care costs [3], notwithstanding decreased rates of parental employee absenteeism and significantly decreased environmental burden for disposal of formula cans and bottles [4].

For optimal growth and development, the World Health Organization (WHO) recommends exclusive breastfeeding for the first six months of life, starting in the first half hour after delivery [5]. However, in most countries, the proportion of mothers who exclusively breastfeed their babies up to 6 months remains low [6]. The resultant child malnutrition has alarming long-term implications, particularly in low and middle-income settings where it has been associated with decreased schooling and economic productivity later in life [7].

Understanding the context-specific patterns and determinants of breastfeeding practices is necessary to ensure successful breastfeeding promotion strategies [8]. As such, a large number of studies have evaluated the socio-demographic, psychosocial, medical and policy factors associated with breastfeeding initiation and maintenance [9]. For example, younger and less educated women have been shown to be less likely to breastfeed in Europe [10]. Maternal employment has been consistently cited as an obstacle to breastfeeding [11]. Maternal breastfeeding attitudes, including self-efficacy and intent to breastfeed have been associated with increased breastfeeding duration in the United States [12]. Acute mental and physical stress among breastfeeding mothers may impair lactogenesis [13]. In particular, self-reported postpartum depression or anxiety among mothers has been found to be significantly negatively associated with breastfeeding at 6 months postpartum [14]. Most studies on determinants of breastfeeding have been conducted in high-income countries and as such, findings may not be applicable to low and middle-income countries. Among the latter, the Middle East region has been largely under-represented until recent years.

Although various breastfeeding promotion initiatives have been established in Lebanon, breastfeeding rates remain low. According to the 2006 Pan Arab Family Health Survey (PAPFAM), while up to 89% of infants are ever breastfed, only 24% of infants below 4 months are exclusively breastfed. The average duration of breastfeeding is 9 months [15]. One study conducted in Beirut found that the breastfeeding rate was 56% at 1 month and 24% at 4 months [16]. Similarly, a national survey conducted in 2004 reported breastfeeding rates as low as 52% at 1 month despite extremely high breastfeeding initiation rates [17]. In the same survey, only 18% of mothers breastfed within 30 minutes after delivery. Hospital policies, employment and type of delivery have all been shown to significantly affect breastfeeding practices in Lebanon [18,19]. One study showed a significant effect of religion and pediatrician gender on breastfeeding [16] while another documented common cultural beliefs that may discourage breastfeeding [20].

To date, studies that have addressed possible predictors of breastfeeding behavior in Lebanon remain scarce. This paper aims to assess the prevalence of breastfeeding in Beirut and determine the factors that impact breastfeeding behavior in this population. A better understanding of these factors would contribute to a larger academic understanding of the breastfeeding ecosystem in Lebanon, assist breastfeeding promotion agencies and practitioners to implement successful strategies, and catalyze the development of effective breastfeeding-friendly policies.

## Methods

### Setting

Lebanon is a small middle-income country on the Eastern Mediterranean, with an estimated population of 4 million and a fertility rate of 1.7 [21]. Its population is highly urbanized (87%) and highly literate (87%) [21]. Its capital city, Beirut, has an estimated population of 361 366 (9.6% total population) and an average household size of 3.75 [22]. Only 17% of all women in the city are part of the labor force [15]. The study was carried out in Beirut and its close suburbs. Close to 100% of all births in Beirut are attended by a skilled birth attendant [15]. All hospitals (26 private and 1 public) with maternity wards in the city and its close suburbs were considered eligible for enrollment. Twenty-three (22 private, 1 public) hospitals agreed to participate. None of these hospitals implement the WHO/UNICEF Baby-Friendly Hospital Initiative.

### Design and data source

This was a secondary analysis of data from a randomized control trial (RCT) assessing the impact of a 24-hour hotline service and postpartum support film on postpartum stress among first-time mothers [unpublished data - Osman]. First-time mothers were randomized according to a computer-generated random list into one of four groups (postpartum support film, hotline service, postpartum support film and hotline service, or control group that received neither). A randomized controlled single-blind design was used. The postpartum support film was recorded on a DVD, the hotline service number was marked on a card and the control group entailed a music CD. All materials were placed in a hard DVD cover and in consecutively-numbered opaque envelopes that looked and felt the same. These were handed to every mother by recruiters who were blinded to their contents.

Based on power calculations for the original RCT (see below), a total of 751 primiparous women were contacted: 119 were excluded and 80 refused to participate. 552 women received the baseline interview.

The study was conducted between March and July 2009. Trained interviewers conducted a baseline and postpartum assessment of healthy first-time mothers at 1–3 days and 8–12 weeks post-delivery respectively.

Baseline data was collected daily over a 7-week period by 8 recruiters who included midwives and public health graduate students. Recruiters visited participating hospitals daily at same time (8-10am) to include all primipara deliveries that met study inclusion criteria. At each hospital visit, the recruiter reviewed the list of deliveries in the last 24 hours and visited every woman who met the inclusion criteria in her room. Written informed consent was obtained from each prospective participant. A 3-minute baseline interview was conducted to gather information about the woman’s socio-demographic status, her health and that of her baby as well as her pregnancy, delivery experience and intent to breastfeed.

Fourteen assessors were trained to conduct the postpartum interview. Interviews were conducted face to face at women’s homes when possible. Telephone assessments were conducted when assessors were unable to visit women at home. The postpartum interview lasted between 30 to 50 minutes. It included questions about general health, infant health and care, breastfeeding attitude and behavior, marital life, employment and perceptions of self-efficacy and social support. In addition, screening tests for postpartum anxiety and stress as well as postpartum depression were conducted.

### Eligible participants

All first time mothers delivering within participating hospitals were eligible for inclusion in the study. They were excluded if they had any of the following characteristics: 1) multiple or complicated gestations, 2) chronic diseases requiring daily management such as cardiovascular diseases, hypertension, diabetes, or thyroid diseases, 3) infant in the neonatal intensive care unit, or 4) they were planning on leaving Lebanon before the time of assessment.

### Variables

The outcome of this study was exclusive breastfeeding at 8 to 12 weeks postpartum. It was categorized into exclusive breastfeeding and non-exclusive breastfeeding (consisting of complementary feeding and infant milk formula). As per the WHO definition, exclusive breastfeeding was defined as giving no other food or drink, not even water, except breast milk (including milk expressed or from a wet nurse) but allows the infant to receive oral rehydration solution (ORS), drops and syrups (vitamins, minerals and medicines).

Based on a thorough literature review, a set of factors was selected to assess their associations with the outcome (Table 1). These included the woman’s socio-demographic characteristics (age, education, employment status, household income), pregnancy and delivery indicators (planned pregnancy, mode of delivery, health attitude and behavior, postpartum health and postpartum social support, the infant’s characteristics and the intervention arm allocation. Postpartum health included three measures of psychological health. Stress was measured by the Arabic Cohen Perceived Stress Scale- PSS-10, a validated scale in Arabic [23]. Each item was measured on a 4-point scale. The total score was dichotomized based on the median value. Postpartum depression was assessed using The Arabic Edinburgh Postnatal Depression Scale (EPDS) [24]; women who scored above a threshold of 12/13 in the EPDS were considered to have postpartum depression. Anxiety was measured by the Spielberger State-Trait Anxiety Inventory (SSTAI); this questionnaire had been previously translated into Arabic and validated [25]. A total score was calculated and then regrouped into two categories.

**Table 1:** Factors tested for their association with exclusive breastfeeding

**Woman socio-demographic characteristics**	Age, education, employment status, household income
**Pregnancy and delivery indicators**	Planning of pregnancy, gestational age, mode of delivery, companionship at delivery, delivery experience, holding baby in first 30 min, holding baby in first 2 hours, rooming-in most of the time
**Woman health attitude and behavior**	Smoking, weight gain in pregnancy, intention to breastfeed exclusively
**Woman postpartum health**	Having a health problem, depression, anxiety level, stress level
**Baby characteristics**	Having a health problem, baby behavior
**Social support**	Perceived social support, main emotional support
**Intervention arms**	Video, hotline, both video and hotline

### Sample size

Sample size was calculated based on the aim of reducing the PSS-10 mean by 4 points in the original RCT. The mean score for the PSS-10 was found to be 18.3, with a standard deviation of 4.9 in the validation study among postpartum women in Lebanon. Based on the assumption that 50% would watch the film, the mean for the intervention group was considered to be 16.3. Therefore, 126 women were needed in each arm with an alpha of 0.05 and a power of 90%. Accounting for 10% loss to follow-up, 140 women were needed for each arm. Although these power calculations were conducted for the original RCT, they are similarly adequate for this cross-sectional study given the multiple outcomes tested and allowed for.

### Analysis

The statistical package for social sciences (SPSS) version 16 was used. Descriptive statistics were done to examine variability of all chosen factors and to decide on bracketing (Table 1). The two continuous scales, PSS-10, SSTAI were dichotomized as they exhibited a non-linear association with the outcome. They were dichotomized based on their median. Bivariate analyses were performed to assess the crude associations between the chosen factors and exclusive breastfeeding. Chi square tests were performed and p-values reported. Variables that were associated with the outcome with p<0.1 were included in the final multivariate logistic regression. Potential collinearity of independent variables was then examined by computing variance inflation factor (VIF) scores for pairwise associations. All VIF scores were <4, indicating probable absence of collinearity. Unadjusted and adjusted odds ratios (OR) were reported with their 95% confidence intervals and p-values.

### Ethical considerations

The original study was approved by the American University of Beirut Institutional Review Board in accordance to the National Institute of Health guidelines. The original trial was registered with clinicaltrials.gov (identifier # NCT00857051) on March 5, 2009. Applications were also submitted and approved by ethical review boards of participating hospitals when required.

## Results

### Sample characteristics

A total of 751 primiparous women were approached to participate with a 74% enrollment rate (119 were excluded and 80 refused to participate). There were no significant differences between the socio-demographic characteristics of women who participated and those who refused (data not shown). Of the 552 women who received the baseline assessment, 452 (82%) completed the postpartum questionnaire and 100 (18%) were lost to follow-up. Of the 452 who completed the postpartum assessment, 325 (72%) had a face-to-face interview and 127 (28%) completed it over the telephone. There were no significant differences between the socio-demographic characteristics of women who were assessed and those who were lost to follow up.

The majority of women had secondary education and above (70.8%), with slightly over half of them (51.2%) having reached university. Around sixty percent were unemployed and reported a household monthly income of over 1 million Lebanese pounds (equivalent to 660 US Dollars). Most women did not report any health problem in the postpartum period. However, the prevalence of stress, anxiety and depression symptoms was relatively high (50%, 47.7%, and 33.6% respectively). The large majority of newborns was term (91.4%) and close to half (46.3%) was delivered by C-section. The largest percent of mothers intended to exclusively breastfeed (87.1%) (Table 2).

**Table 2:** Factors related to exclusive breastfeeding

	**Total N (%)**	**Exclusive breastfeeding**		**p-value**
		**Yes**	**No**	
**I. Woman socio-demographic characteristics**				
Age				0.003
<20	46 (10.3%)	14 (30.4%)	32 (69.6%)	
20-24	114 (25.4%)	47 (41.2%)	67 (58.8%)	
25-29	157 (35.0%)	33 (21.0%)	124 (79.0%)	
30-34	88 (19.6%)	19 (21.6%)	69 (78.4%)	
≥ 35	43 (9.6%)	11 (25.6%)	32 (74.4%)	
Education				0.280
Intermediate and below	130 (29.2%)	41 (31.5%)	89 (68.5 %)	
Secondary	87 (19.6%)	27 (31.0%)	60 (69.0%)	
University	228 (51.2%)	56 (24.6%)	172 (75.4%)	
Employed				0.000
Yes	167 (37.9%)	23 (13.8%)	144 (86.2%)	
No	274 (62.1%)	98 (35.8%)	176 (64.2%)	
Monthly Household income (Lebanese pounds)				0.019
< 1 million	180 (39.8%)	60 (33.3%)	120 (66.7%)	
1-2 millions	162 (35.8%)	44 (27.2%)	118 (72.8%)	
> 2 millions	110 (24.3%)	20 (18.2%)	90 (81.8%)	
**II. Pregnancy and delivery indicators**				
Planned pregnancy				0.068
Yes	306 (68.0%)	91 (29.7%)	215 (70.3%)	
No	144 (32.0%)	31 (21.5%)	113 (78.5%)	
Gestational age				0.036
Preterm	36 (8.6%)	4 (11.1%)	32 (88.9%)	
Term	384 (91.4%)	104 (27.1%)	280 (72.9%)	
Mode of delivery				0.016
Vaginal	240 (53.7%)	78 (32.5%)	162 (67.5%)	
C-section	207 (46.3%)	46 (22.2%)	161 (77.8%)	
Had a companion at delivery				0.937
Yes	181 (40.1%)	49 (27.1%)	132 (72.9%)	
No	270 (59.9%)	74 (27.4%)	196 (72.6%)	
Delivery experience				0.186
As she expected or better	32 (7.1%)	12 (37.5%)	20 (62.5%)	
Worse than she expected	420 (92.9%)	112 (26.7%)	308 (73.3%)	
Held baby in first 30 min				0.885
Yes	223 (49.4%)	62 (27.8%)	161 (72.2%)	
No	228 (50.6%)	62 (27.2%)	166 (72.8%)	
Held baby in first 2 hours				0.385
Yes	320 (71.0%)	91 (28.4%)	229 (71.6%)	
No	131 (29.0%)	32 (24.4%)	99 (75.6%)	
Rooming-in most of the time				0. 477
Yes	257 (57.0%)	74 (28.8%)	183 (71.2%)	
No	194 (43.0%)	50 (25.8%)	144 (74.2%)	
**III. Woman health attitude and behavior**				
Smoking				0.186
Ever	157 (35.2%)	37 (23.6%)	120 (76.4%)	
Never	289 (64.8%)	85 (29.4%)	204 (70.6%)	
Weight gain in pregnancy				0.105
<= 15 kg	279 (62.1%)	69 (24.7%)	210 (75.3%)	
>= 16 kg	170 (37.9%)	54 (31.8%)	116 (68.2%)	
Intention to breastfeed exclusively				0.000
Yes	390 (87.1%)	117 (30.0%)	273 (70.0%)	
No	58 (12.9%)	4 (6.9%)	54 (93.1%)	
**IV. Woman postpartum health**				
Had a health problem				0.292
Yes	83 (18.4%)	19 (22.9%)	64 (77.1%)	
No	367 (81.6%)	105 (28.6%)	262 (71.4%)	
Depressed				0.135
Yes	152 (33.6%)	35 (23.0%)	117 (77.0%)	
No	300 (66.4%)	89 (29.7%)	211 (70.3%)	
Anxiety level				0.133
Below median	235 (52.3%)	72 (30.6%)	163 (69.4%)	
Above median	214 (47.7%)	52 (24.3%)	162 (75.7%)	
Stress level				0.092
Below median	226 (50.0%)	70 (31.0%)	156 (69.0%)	
Above median	226 (50.0%)	54 (23.9%)	172 (76.1%)	
**V. Baby characteristics**				
Had a health problem				0.003
Yes	130 (28.8%)	23 (17.7%)	107 (82.3%)	
No	322 (71.2%)	101 (31.4%)	221 (68.6%)	
Baby behavior				0.382
Easy / normal baby	164 (36.3%)	41 (25.0%)	123 (75.0%)	
Fussy baby	288 (63.7%)	83 (28.8%)	205 (71.2%)	
**VI. Social support**				
Perceived social support				0.897
Valuable	294 (65.9%)	81 (27.6%)	213 (72.4%)	
Not valuable	152 (34.1%)	41 (27.0%)	111 (73.0%)	
Main emotional support				0.012
Mother	185 (40.9%)	39 (21.1%)	146 (78.9%)	
Other	267 (59.1%)	85 (31.8%)	182 (68.2%)	
**VII. Intervention arms**				
				0.119
Video	127 (28.1%)	35 (27.6%)	92 (72.4%)	
Hotline	121 (26.8%)	41 (33.9%)	80 (66.1%)	
Both	101 (22.3%)	28 (27.7%)	73 (72.3%)	
Neither	103 (22.8%)	20 (19.4%)	83 (80.6%)	

* all figures do not add up to original sample size due to missing values.

Most (67%) mothers were breastfeeding at 8–12 weeks postpartum. Only 27.4% of mothers were exclusively breastfeeding at that time, 39.6% were giving both breast milk and infant formula while 33% were giving infant formula only.

### Bivariate analysis

Various factors were tested for their association with exclusive breastfeeding (Table 2). These were categorized into seven groups: socio demographic characteristics, pregnancy and delivery indicators, women health attitude and behavior, postpartum health, infant characteristics, and social support. The results of the bivariate analysis are shown in Table 2. Maternal stress and whether the infant was the result of a planned pregnancy were factors marginally associated with exclusive breastfeeding (*p*-value= 0.092 and 0.068, respectively). Factors significantly associated with exclusive breastfeeding included maternal age, employment and monthly household income, gestational age and mode of delivery, intention to breastfeed at the time of delivery, baby’s health and main source of emotional support for the new mother.

41.2% of mothers whose ages were between 20 and 24 exclusively breastfed their infant (*p*-value= 0.003) compared with 30.4% of younger mothers. Only 13.8% of working mothers exclusively breastfed compared with 35.8% of non-working mothers (*p*-value< 0.001). Interestingly, 18.2% of those whose monthly household income was more than 2 million Lebanese Pounds (around 1,333 US Dollars) exclusively breastfed their infant compared with 33.3% of those whose monthly income was below 1 million Lebanese Pounds (around 600 US Dollars) (*p*-value= 0.019). Only 11.1% of mothers of preterm infants exclusively breastfed compared with 27.1% of mothers of term infants (p-value=0.036). 22.2% of those who underwent a C-section exclusively breastfed compared with 32.5% of those who underwent a vaginal delivery (*p*-value= 0.016).

Intention to exclusively breastfeed was strongly associated with exclusive breastfeeding (*p*-value< 0.001). 30% of women who intended to exclusively breastfeed at the time of delivery were doing so, compared to 6.9% of those who did not intend to. A significantly higher proportion of mothers who did not report any health problems in their newborn breastfed exclusively compared to those who reported that their babies had health problems (31.4% compared with 17.7% respectively with *p*-value= 0.003).

21.1% of women who identified their mothers as their main source of emotional support exclusively breastfed compared with 31.8% of women who reported their husbands or others as their primary source of emotional support (*p*-value= 0.012).

### Multivariate analysis

Maternal work, intention to breastfeed, pregnancy planning, source of emotional support and the intervention arms were significantly associated with exclusive breastfeeding (Table 3). Non-working mothers were more likely to exclusively breastfeed than working mothers (OR=3.92; p-value<0.001). Furthermore, mothers who intended to breastfeed exclusively at the time of delivery were more likely to do so compared with those who did not intend to (OR=3.28; p-value=0.043). Women who had planned their pregnancy were more likely to exclusively breastfeed compared with those who had not (OR=2.42, p-value=0.010). Women who did not identify their own mothers as their primary source of emotional support were more likely to exclusively breastfeed (OR = 1.87, p-value=0.039). Compared to women who did not receive the support film or used the hotline, women in any of the three intervention arms were more likely to exclusively breastfeed, with those receiving both the video and the hotline interventions being the most likely to exclusively breastfeed (OR=2.55, p-value=0.044; OR=3.87, p-value=0.004 and OR=4.13, p-value=0.003 respectively).

**Table 3:** Adjusted and unadjusted odds of exclusive breastfeeding with their 95% confidence intervals by selected demographic, psychosocial and health factors

Variable*	**Unadjusted OR [95% CI]**	**Adjusted OR [95% CI]**	***p*-value**
**I. Woman socio-demographic characteristics**			
Age (<20)			
20-24	1.60 [0.77-3.33]	1.54 [0.57-4.19]	0.398
25-29	0.61 [0.29-1.27]	0.91 [0.32-2.60]	0.863
30-34	0.63 [0.28-1.41]	0.72 [0.22-2.34]	0.580
≥ 35	0.79 [0.31-2.00]	1.11 [0.32-3.89]	0.868
Woman’s work (employed)			
Unemployed	3.49 [2.11-5.78]	3.92 [1.93-7.94]	0.000
Income (>2 millions)			
<1 million	2.91 [1.46-5.82]	1.05 [0.44-2.51]	0.918
1-2 millions	1.93 [0.93-3.99]	1.17 [0.51-2.68]	0.704
**II. Pregnancy and delivery indicators**			
Planned pregnancy (No)			
Yes	1.54 [0.97-2.46]	2.42 [1.24-4.73]	0.010
Gestational age (Preterm)			
Term (≥ 37 weeks)	2.97 [1.03-8.61]	2.90 [0.80-10.51]	0.104
Mode of delivery (C-section)			
Vaginal	1.69 [1.10-2.58]	1.31 [0.73-2.35]	0.361
**III. Woman health attitude and behavior**			
Breastfeeding intention (Intention to exclusively breastfeed)			
No intention to exclusively breastfeed	5.79 [2.05-16.34]	3.28 [1.04-10.31]	0.043
**IV. Woman postpartum health**			
Stress level (Below median)			
Above median	0.70 [0.46-1.06]	1.32 [0.74-2.36]	0.339
**V. Baby characteristics**			
Baby health problem (Yes)			
No	2.13 [1.28-3.53]	1.88 [0.98-3.58]	0.056
**VI. Social support**			
Main emotional support (Mother)			
Other	1.75 [1.13-2.71]	1.87 [1.03-3.40]	0.039
**VII. Intervention arms (Music)**			
Video	1.58 [0.85-2.95]	2.55 [1.03-6.32]	0.044
Hotline	2.13 [1.15-3.94]	3.87 [1.55-9.63]	0.004
Both video and hotline	1.59 [0.83-3.06]	4.13 [1.61-10.60]	0.003

* The variables in parentheses indicate reference categories.

## Discussion

This study confirms the low prevalence of exclusive breastfeeding at 8–12 weeks postpartum in a representative sample of women in Beirut. The large majority of the mothers in this study were not exclusively breastfeeding at 8–12 weeks postpartum. Prevalence of exclusive breastfeeding at 6 months postpartum is likely to be even lower. This finding is similar to findings from other developing countries demonstrating the low adherence to the WHO breastfeeding guidelines [26,27]. In Lebanon, past research has cited detrimental cultural beliefs (insufficient milk, “bad” colostrum) [19], unfavorable hospital policies [20] and discouraging physician advice as potential barriers to exclusive breastfeeding and catalysts to breastfeeding cessation.

In this study, almost all mothers intended to exclusively breastfeed, similar to observations in neighboring countries such as Syria and Jordan [28]. The fact that women who intended to exclusively breastfeed at the time of delivery were more than three times more likely to do so than those who did not is not surprising. There is extensive evidence on the effectiveness of the Theory of Planned Behavior (TPB) in predicting breastfeeding practices and its applicability in cross-cultural settings [29]. According to TPB, intent is an immediate antecedent to behavior change. The large proportion of women who intended to exclusively breastfeed seems to imply that these women foster positive breastfeeding beliefs, attitudes and norms. It is noteworthy, however, that more than two thirds of the women who intended to exclusively breastfeed were not doing so at follow up. Similarly, a prospective study in Canada found that most of the 189 first-time mothers who had planned to exclusively breastfeed for 6 weeks did not meet their goals [30]. These findings suggest there are significant mediators beyond individual intent that could alter breastfeeding behavior. Interestingly, 6.9% of mothers who did not intend to exclusively breastfeed ultimately did so. Although it is not clear why this occurred, further investigation of the factors that resulted in this change of heart may uncover important elements that could prove useful for the promotion of exclusive breastfeeding.

The large C-section rate found in this study was similar to C-section rates in Beirut reported in other studies [31-33]. Although mode of delivery was not found to be a significant predictor of exclusive breastfeeding in the regression model, the large majority of women who did not exclusively breastfeed had had a C-section. There is a wealth of evidence suggesting C-sections are detrimental to breastfeeding [34]. In Lebanon, this is particularly relevant, as elective C-sections have increased at an alarming rate over the past years and the policy environment continues to encourage them [35]. In our study, however, C-sections were not significantly associated with exclusive breastfeeding. One possible explanation could be that a large number of C-sections performed at hospitals in Beirut are elective- or not medically indicated. As a result, many mothers who deliver by C-sections in Beirut are healthy and able to initiate breastfeeding faster than mothers who have had a C-section for a medical reason and may be more ill. One of the strongest associations found in this study was between work and exclusive breastfeeding. Working mothers were much less likely to exclusively breastfeed compared with their non-working counterparts. This finding is of special relevance as first-time mothers are increasingly part of the labor force in Lebanon [36]. The possible negative effect of employment on exclusive breastfeeding documented in this study has been replicated in different settings [37]. In Lebanon, the work environment for women presents significant barriers to breastfeeding. A recent study of 802 Lebanese women found that the 49-day maternity leave was considered insufficient for most mothers and that more breastfeeding-friendly policies are needed in the workplace, including flexible nursing times and adequate nursing facilities [38]. Such interventions have been implemented successfully in Iran for example, where exclusive breastfeeding rates are among the highest in the region [39]. The economic, political and public health implications of the association between working mothers and breastfeeding remain largely unexplored in the Lebanese context. Our finding could serve as a stepping-stone for further research into that particular area. As more Lebanese women enter the work force, awareness efforts may need to target employers and policymakers. Pediatricians should identify working mothers as a “high risk” group and counsel them accordingly.

The hotline service instituted as part of the original RCT also had a significant impact on exclusive breastfeeding. Women who used the hotline were almost four times as likely to exclusively breastfeed. This may indicate the need for more awareness and/or support to breastfeeding mothers. In today’s ever-growing time constraints, our result emphasizes the value, for women’s health practitioners, of simply taking the time to answer a question. Given that the hotline issues addressed were not restricted to breastfeeding, it would be interesting to investigate whether the impact of a “breastfeeding hotline” would be greater. If so, a national hotline could prove to be a cheap and feasible way to raise breastfeeding rates.

### Limitations

The restriction of participants to healthy first-time mothers delivering in Beirut is one limitation of this study. The conclusions reached may not be applicable to the larger Lebanese population, especially given the likely differences in breastfeeding practices among urban and rural mothers [40]. Furthermore, different factors may come into play with ill mothers and children. It is not unreasonable to suggest that those groups may be at increased risk of formula feeding and/or early complementary feeding given the added stressors on the mother and family. Past exposure to breastfeeding within the mother’s social networks may affect breastfeeding behavior; these associations were not captured in this study. The independent variables assessed were based on self-reports that are subjective by definition. Exclusive breastfeeding at 8–12 weeks was chosen as an outcome rather than the standard 6 months defined by the WHO because the study was not originally designed to capture breastfeeding outcomes. Its results are nested within a larger RCT that aimed at assessing postpartum stress and depression rates at 8–12 weeks postpartum. As with any longitudinal study, the potential for recall bias is always present; however, the short recall period makes this unlikely to have caused significant change in results. Finally, the percent of mothers who declined to participate (26%) and those who were lost to follow up (18%) could have introduced another bias if these non-participating groups were systematically different from mothers who were included in this study. However this is unlikely given these groups were not statistically different from the participating mothers in terms of socio-demographic characteristics.

## Conclusions

The proportion of healthy first-time mothers who exclusively breastfeed in Beirut is extremely low. This evidence is a timely reminder that much has yet to be done to promote breastfeeding in Lebanon. Understanding its determinants is a first step towards developing successful interventions and policies. This study has found that maternal employment status, intent to breastfeed and baby’s gestational age and physical health are significantly associated with exclusive breastfeeding. The diversity of these factors dictates an ecological approach to breastfeeding promotion. Multi-faceted interventions across different levels of the maternal-child pair’s ecosystem are likely to be the most effective. Beyond the epidemiology of breastfeeding, further research is needed to assess which multi-level strategies most effectively raise breastfeeding outcomes.

## Competing interests

The authors declare that they have no competing interests.

## Authors’ contributions

HH contributed to the literature review and interpretation of data. MC contributed to the design, analysis and interpretation of data. MS contributed to data collection, management and analysis. RC contributed to the literature review and data analysis. HO contributed to the conceptualization of the study design, analysis and interpretation of data. All authors contributed to the writing of the manuscript. All authors read and approved the final manuscript.

## Pre-publication history

The pre-publication history for this paper can be accessed here:

http://www.biomedcentral.com/1471-2458/13/702/prepub
